# Draft Genome Sequence of Bacillus sp. Strain UFRGS-B20, a Hydrocarbon Degrader

**DOI:** 10.1128/genomeA.00052-18

**Published:** 2018-02-22

**Authors:** Patricia Dörr de Quadros, Roberta Fulthorpe, Rosemary Saati, Vanessa Cerqueira, Fátima Menezes Bento

**Affiliations:** a Department of Environmental Microbiology, Federal University of Rio Grande do Sul, Porto Alegre, Rio Grande do Sul, Brazil; b Department of Physical and Environmental Sciences, University of Toronto, Toronto, Ontario, Canada

## Abstract

Bacillus sp. strain UFRGS-B20 was isolated in 2012 from Brazilian land-farming soil contaminated with petrochemical oily sludge. This strain was subjected to hydrocarbon biodegradation tests, showing degradation rates of up to 60%. Here, we present the 6.82-Mb draft genome sequence of the strain, which contains 2,178 proteins with functional assignments.

## GENOME ANNOUNCEMENT

Bacillus sp. strain UFRGS-B20 was isolated from soil contaminated with petrochemical oily sludge (bioremediation land-farming site) in South Brazil and cultured in Luria-Bertani (LB) medium containing different concentrations of sterilized oily sludge. It increased biomass significantly (Tukey test, α < 0.01) in the presence of 1% oily sludge compared to the no-oily-sludge treatment (just LB medium). This strain was also tested in oily sludge-contaminated soil microcosms, where it degraded up to 60% of polycyclic hydrocarbons (1). This strain was proven to degrade polycyclic aromatic hydrocarbons in liquid and solid environments, and it has the genetic potential to be used in the bioremediation of contaminated soils or water.

The genomic DNA of Bacillus sp. strain UFRGS-B20 was isolated using the QIAamp DNA minikit (Qiagen), and it was used to generate the PacBio whole-genome shotgun library of sheared long inserts. The sequencing was performed using Pacific Biosciences (PacBio) RS II sequencing technology at McGill University and the Génome Québec Innovation Centre (30× coverage). The sequences were treated and assembled using miniasm (2) in PATRIC version 3.5.2 (3), and the genome annotation was performed using PATRIC. The *N*_50_ and *N*_75_ values of the assembly were 278,944 and 126,449 bp, respectively. The largest contig was 1,546,980 bp. The draft genome showed 88.07% similarity with that of Bacillus cereus (NCBI accession number GCA_000007825, NCBI assembly number ASM782v1) and 87.47% similarity with that of Bacillus thuringiensis (GCA_002243685, ASM224368v1), so its classification falls into an undefined species. The sequences consisted of 6,827,566 bp, with a GC content of 35.78%, distributed within 59 contigs. No plasmid sequences were detected.

Bacillus sp. strain UFRGS-B20 contains genes encoding enzymes related to 17 pathways of xenobiotic biodegradation and metabolism, including hydrolase aldolase (AI-2) LsrF (bisphenol degradation), succinate dehydrogenase, 3-hydroxyacyl-coenzyme A (3-hydroxyacyl-CoA) dehydrogenase, methyltransferases, alcohol and aldehyde dehydrogenases, and hydrolases involved in naphthalene, anthracene, and 1- and 2-methylnaphthalene degradation; (*S*)-2-haloacid dehalogenase, cytochrome P450 102A3, monooxygenases, acid and alkaline phosphatases, acetamidases, nitric oxide synthase oxygenase, and oxygen-insensitive NAD(P)H nitroreductase; and 3-ketoacyl-CoA thiolase, *N*-acetyltransferase, ureases, allophanate hydrolase, gluconolactonase, and enoyl-CoA hydratases. Furthermore, we also detected 31 genes coding for methyltransferases, 8 genes coding for antibiotic resistance, and 13 genes coding for efflux pump genes. Bacillus sp. strain UFRGS-B20 also contains genes related to the biosynthesis of secondary metabolites of biotechnological importance, such as puromycin, tetracycline, novobiocin, streptomycin, terpenoid backbone, diterpenoids, carotenoids, zeatin, phenylpropanoids, flavonoids, anthocyanin, flavone, flavonol, stilbenoids, diarylheptanoids, gingerol, isoquinoline alkaloids, tropane, piperidine alkaloids, betalain, and insect hormone, as well as for biosynthesis of polyketides and nonribosomal peptides such as ansamycins, siderophores, and type II polyketide backbone and products. Finally, this strain is involved in the metabolism of C_21_-steroid hormones, porphyrin, and chlorophyll.

### Accession number(s).

This whole-genome shotgun project has been deposited at GenBank under accession number POCE00000000.
